# Sensitivity to the role of an animated agent from observed interactions in newborn chicks (*Gallus gallus*)

**DOI:** 10.1098/rsos.210020

**Published:** 2023-10-25

**Authors:** P. De Roni, A. Geraci, F. Simion, L. Regolin

**Affiliations:** ^1^ Department of Developmental Psychology and Socialisation, University of Padova, 35131 Padua, Italy; ^2^ Department of General Psychology, University of Padova, 35131 Padua, Italy; ^3^ Department of Social and Educational Sciences of the Mediterranean Area, University for Foreigners of Reggio Calabria, 89125 Reggio Calabria, Italy

**Keywords:** *Gallus gallus domesticus*, social role, social hierarchy, social development, early preference, avian cognition

## Abstract

Few month old human infants are able to detect the social roles of artificial agents and consistently choose the object behaving as ‘approacher’ rather than ‘repulser’. This preference has been considered evidence of a pre-linguistic and pre-cultural origin of the social mind. Similar preferences have not been described in other species, though comparative data could help clarify the nature of this phenomenon and its evolutionary origin. In this study, we investigated sensitivity to the social role of an artificial agent in domestic chicks. Birds offer an excellent model to study the evolutionary roots of cognitive abilities, since they separated from mammals over 300 Ma. Moreover, the investigation of newly hatched chicks allows control for previous experience. After being exposed to computer-presented animations depicting an interaction among two agents, chicks underwent a free choice test among those same objects. While no initial evidence of a clear preference emerged from the planned analysis, chicks in the experimental condition showed a preference for the ‘approacher’ when controlling for side bias, mirroring human infants behaviour. This suggests the existence of an early ability to discriminate agents from their interactions, independent from any social experience

## 1. Introduction

Newborn chicks (*Gallus gallus*) are vunerable when facing potential predators, and rely for protection on the ability to maintain physical proximity to the mother hen and their siblings [1,2]. Chicks can promptly learn to recognize and remember these ‘good characters’ and tell them apart from unknown and potentially dangerous conspecifics [3–5]. In fact, both chicks and adults behave aggressively towards stranger conspecifics. Chicks can discriminate familiar from unfamiliar individuals, and most likely they can also discriminate individual animals based on visual cues ([4,6,7], see [8] for an in-depth overview on the species' behaviour).

We know that very early in life chicks can form a stable social hierarchy [9] for which individual recognition is necessary as well as the ability to rank other individuals. Moreover, females seem to form larger groups with stricter hierarchies compared with males [10]. This could reflect in a higher ability of females to detect third-party social roles. Ranking can take place by engaging directly with others or by exploiting indirect information (i.e. observed interactions among other chickens). In fact, chickens can assess others’ social rank when witnessing a fight among a stranger and a familiar individual which social rank is known (in relation to the own rank) [11]. The ability to use indirect information to deduce the social rank is highly advantageous as it may avoid having to directly confront with every other individual in the group, thereby saving energy and avoiding injuries. Such ability is probably widespread in vertebrates as it is widely described in fish species [12–14], as well as in pinyon jays (*Gymnorhinus cyanocephalus*) [15], chimpanzees (*Pan troglodytes*) [16] and pigtailed macaques (*Macaca nemestrina*) [17]. Ritualized behavioural interactions between dominant and inferior pack members are also observed in wolves, as a way to formalize social hierarchy without engaging in an actual fight [18,19].

Newborn domestic chicks exhibit impressive social predispositions towards naturalistic objects (reviewed in [8,20]). These may be observed even when chicks deal with non-naturalistic objects. In fact, it is well known that filial *imprinting* [21–23] also takes place towards artificial objects: from a stuffed animal to geometrical figures or patterns of shapes [24–26]. Precocial birds can even extract relational properties of groups of objects such as ‘same colour’ or ‘same shape’ and generalize such properties to new sets of different colour or shape [27,28].

Chicks’ social predispositions are often assessed through the choice between stimuli displaying agent-like features versus unnatural and inanimate stimuli, e.g. the preference for biological versus rigid motion of point light displays [29], or the preference for self-propelled objects versus objects passively displaced owing to physical contact [30,31]. To our knowledge, the preference among two social agents based on qualitative differences in their ‘behaviour’ has not yet been investigated. This was precisely the aim of the present study. Chicks were exposed to computer animations depicting the interaction among two self-propelled agents moving towards each other, with one of them, the ‘repulser’ agent, repetitively hitting the ‘approacher’ agent (the terminology was taken from [32], who used the same paradigm and stimuli to test preverbal infants' preferences).

Geraci *et al*. [32], presented four and eight month old infants with a simple interaction involving two agents: the ‘repulser’ moved towards and pushed the ‘approacher’, which reacted by approaching the repulser without contacting it. Older infants looked longer at the approacher than at the repulser, whereas younger infants exhibited no preference.

The capacity to distinguish social roles was largely examined in human infants in laboratory conditions using artificial stimuli, often computer-presented animations. Very early in life infants can understand social interactions, and consistently show a preference for prosocial individuals over antisocial, aggressive or neutral individuals [32–35]. Infants also discriminate agents' social roles on the basis of their interactions [36]. Moreover, four month old infants are sensitive to inequity in the distribution of goods, preferring fair to unfair distributors [32,37,38]. Children, as young as 18 months, spontaneously help unknown individuals, an attitude also described, though to a lesser extent, in juvenile chimpanzees (*P. troglodytes*) [39]. In an aggressive context, infants and toddlers prefer agents intervening in help of others (*protective*) to neutral agents (*non*-*protective*) [40,41], and 21 month olds reward and prefer the defender agent over the non-defender agent following aggression [42]. In the study by Kanakogi *et al*. [35], very young infants (10 month olds) were shown a video representing the interaction between two self-propelled geometrical figures (a triangle and a circle). One of the two figures moved in a way judged (by human adults) as being actively aggressive towards the other, by chasing and repeatedly hitting the second shape. Infants showed a clear preference for the victim over the aggressor in a subsequent manual choice task. The preference dropped to chance level in the control condition, where the two agents had been seen to move independently.

Owing to their limited perceptual and motor abilities at birth, infants can only be tested after some months, once they have been exposed to extensive and uncontrolled amounts of social experience (with their carers and peers). Chickens instead are fully independent from the moment they hatch, providing a valuable model to assess these predispositions under conditions of controlled rearing, hence of controlled previous social experience.

Here, we aimed to extend the study by Kanakogi *et al*. [35] by exposing newborn chicks to videos representing the interaction among two self-propelled objects: a ‘repulser’ and an ‘approacher’ shape (cf. [32]). Chicks then underwent a free choice test among those same two objects. In the control condition, the two objects interacted reciprocally, so that both had equal social role. This condition was designed to control for any low-level preference of the chicks for either agent, since the agents in this condition did not display any clear behavioural difference in their motion patterns. We already know that adult chickens prefer to approach a low-rank to a high-rank individual in a free choice task [43]: chickens were individually positioned in a runway, with the possibility of approaching a second chicken (either high or low ranked). Latency to approach and distance ran were measured, to assess preferences towards the conspecific. Here, we expected that newborn chicks could already discriminate the social relationships between the two artificial agents and show a preference for the ‘approacher’ agent, coherently with that reported for the human infants and in accordance to the preference shown by adult chickens.

After the free choice test, chicks' pecking order was assessed. In fact, we hypothesized that differences in pecking order may affect detection/attribution of social role, or the appearance of a preference for one or the other agent, as described in the next section. We also expected an influence of the chicks’ sex: females, forming larger groups with more complex and stricter hierarchical relations, might show a finer ability to detect or attribute a social role to the animated agents.

The present study aimed at providing some insight on the comparative origin of social preferences and social role attribution. If newborn chicks succeed in discrimination of stimuli featuring different types of actions, some rudimentary ability to extract social information from impoverished visual animations might also be present early in life in non-human species. Chicks will later refine such ability by direct social and perceptual experience [44]. It is important to notice that, to our present knowledge, it is not possible to clarify whether the mechanisms underlying such ability are specific to the social domain, or are—on the contrary—domain-general (i.e. they would also apply to non-social agents). This study does not address, nor answer, this question. A predisposition for detecting specific social roles may be affected by the chick's social status, and could be particularly relevant for subordinates compared with dominant individuals. Being top-ranked, dominant individuals would not benefit much from understanding the social relationships of others. On the contrary, subordinates (mid-ranked individuals in particular) would benefit from detecting and remembering the social status of higher as well as that of lower-ranked individuals. Previous literature reports of hierarchy tests being conducted on chickens from 10 to 20 days of age. The present study constitutes, to our knowledge, the first attempt to assess ranking predispositions, through pecking order, in newborn chicks. Chicks show clear behavioural patterns from the very first days post hatching, manifesting aggressive pecks towards other chicks and differential access to food by the different individuals. It is not yet known whether pecking order, as measured in the newborn chicks, is indicative of the individual rank later in life. This could constitute an interesting issue for future investigations. For the present study, we were interested in assessing the early presence of such aggressive behaviour predispositions and their possible correlations with the chicks' behavioural response in the free choice test.

The preferences described in human infants [35] have been clarified by introducing a neutral agent (not interacting with either the approacher or the repulser). Infants in fact showed both attraction towards the approaching character, which was preferred over the neutral one, as well as dislike of the repulsing character, to which they preferred the neutral agent. In the case a preference is detected at test also in the young chicks, introducing a neutral agent in the exposure animations could enlighten similarities or differences in the underlying mechanisms among the two species. Future studies could include a longitudinal research, that would help us understand the role of social experience on chicks’ preferences and its developmental trajectory in relation to the social rank.

## 2. Hypotheses

H1. Chicks are able to detect the social role of two artificial agents by observing their interaction, and

H1b. Chicks show a stronger preference for the approacher agent over the repulser agent in the experimental (versus control) condition.

H2. The sex of the chicks influences their performance and their preference for a particular agent.

Furthermore, we assessed whether the pecking order of the chicks influences their performance and their preference for a particular agent as an exploratory analysis.

## 3. Material and methods

We measured the time spent next to each of two agents (dependent variable), considering as independent variable the agent's behavioural pattern (repulser’ versus ‘approacher). The sex was considered as a potential moderator, while pecking order was assessed and analysed in an exploratory analysis. We designed the paradigm to control for several potential confounders, as described below, in the sections relative to each experimental phase. We controlled for: (i) side bias; (ii) preference for a perceptual property of the agents (colour); and (iii) effectiveness of the manipulation (control condition) (see §§3.1 and 3.2).

Latency to first approach to the stimuli has been separately analysed as an exploratory variable, as a measure for motivation.

Animals which did not respond to the test (i.e. those who did not approach either agent for the entire duration of the test) were excluded from the data analysis, as specified in §4, and substituted with new subjects. Subjects were newborn domestic chicks (*Ga. gallus*) coming from eggs incubated and hatched in the laboratory. Eggs were provided by a local hatchery (La Pellegrina, San Pietro in Gu, PD, Italy) and incubated in a ‘MG Rurale 70/100’ incubator at controlled temperature and humidity (*t* = 37°C, humidity = 50–60%). Around 4–5 days before hatching eggs were moved from the incubator to a hatching machine (*t* = 37°C, humidity = 50–60%) where they were kept in complete darkness. Both male and female chicks (sexed by the wing at birth) were tested. The subjects were semi-randomly assigned to either the experimental or the control condition, making sure that an equal number of males and females were assigned to each condition. The same experimental procedure was employed, except for the video animation used during the exposure.

### 3.1. General procedure

The procedure consisted of three main phases: (i) exposure, (ii) test, and (iii) social rank (pecking order) evaluation. The first phase consisted of exposing the subjects to a 90 min video animation representing the interaction between two agents. Following the exposure, a 30 min rest was planned, before chicks underwent a free choice test. Finally, 15 min were dedicated to the social rank evaluation. The chicks were then placed back in the rearing room and the experimental procedures were over. Detailed information about each phase is provided below in a dedicated section.

### 3.2. Exposure stimuli

Stimuli consisted of video animations, created using the software Adobe Professional CS6, with a refresh rate of 60 fps, based on Mascalzoni *et al.* [30]. The video used during exposure depicted two social agents of identical shape (squares) and dimension (400 × 400 pixels) but of different colour (red: red/green/blue (RGB) = r255, g0, b0; purple: RGB = r255, g0, b255) interacting with each other. The colours were chosen based on the paper by Mascalzoni *et al*. [30], as these hues do not elicit any preferential choice *per se* in young chicks. During exposure, each chick experienced one video only. The video differed in the experimental and control conditions. In the experimental condition, the video depicted one of the agents (agent A) hitting and pushing the other agent (agent B) (electronic supplementary material, video). Both figures appeared on the screen, briefly stop at the same distance from the centre and then disappeared at the same time. The interaction between the two squares took place at the centre of the screen, to avoid prompting any side biases. To attract the attention of the chick, each collision among the two agents was accompanied by the recorded sound of the tip of a finger tapping on a hard surface. The sound was carefully synchronized with the collision. The side where agent A appeared was alternated during the entire duration of the exposure, looping for 10 min before the side changes (electronic supplementary material, video). The colour (red/purple) and the initial side to which agent A appears (left/right) were counterbalanced between subjects, to control for potential confounders (i.e. side biases or preferences for a specific colour). We planned a second experimental condition, to be run in case the first one did not elicit a clear choice. This option is described at the end of the material and methods section.

In the control condition, each agent equally hit and was hit by the other agent so that both agents had identical roles. In this condition, the same amount of movement as in the experimental condition was preserved, but there was no overall difference in the type of actions, hence in the roles that could be attributed to the two agents. In both the control and the experimental stimuli, one agent initiated the movement and chicks may prefer the first moving agent. In this case, though, we would observe the same preference in both the control and the experimental condition. Any difference between the two conditions must then be attributed as a response to the roles of the agents. In fact, we expected that chicks in the control condition would show no preference for one or the other agent, and choice would drop at chance level.

### 3.3. Test stimuli

The stimuli (video animations) used at test were the same for the experimental and the control condition, and were also created with Adobe Professional CS6 (60 fps). Both agents were presented, but these appeared each in a different half (left–right) of the screen. Agents stayed in the same position, although they kept moving slightly on the spot (by about 15 pixels left–right) to attract the attention of the chicks during the test. To each bird two video animations (3 min long, 60 frames s^−1^) were presented, one after the other. The two animations differed only for the left–right position of the two agents. The order of presentation (which animation is presented first or second) was randomized between subjects.

### 3.4. Exposure

**Figure 1.**
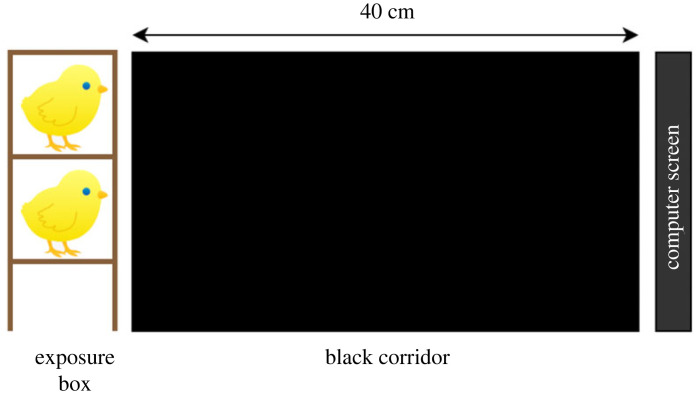
Apparatus for the exposure phase. Chicks were positioned in individual wooden boxes, 40 cm from the screen onto which the video animations were presented.

Between 24 and 48 h from hatching, three chicks of the same sex were transported to the experimental room inside a dark box. They were then positioned inside individual wooden boxes (10 × 10 cm), with a transparent front facing a screen (Samsung, 27″; Model: S27E390H, refresh rate: 75 Hz) (figure 1). The screen, located 40 cm from the chicks, played an animation (see §3.2), running in loop for 90 min (total exposure time). Apart from the light coming from the screen, the experimental room was kept in the dark, with a controlled temperature of 28°C. After the exposure, the three chicks were placed back into the hatchery in total darkness for a rest period of around 30 min (the exact amount of time depending on the chicks' testing order, ranging from 30 min for the first chick tested, to 45 min for the third chick tested). This procedure promotes memory consolidation [45].

### 3.5. Free choice test

**Figure 2.**
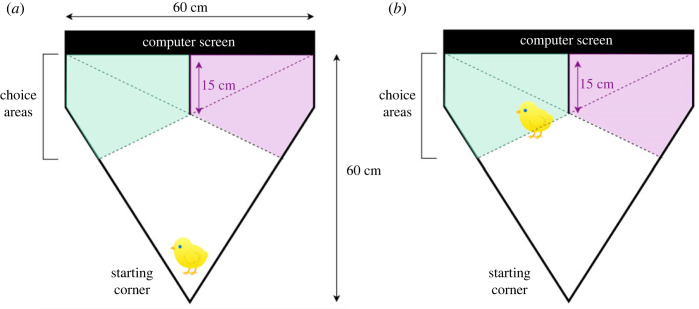
(*a*) Triangular arena used for the free-choice test. On the top side of the triangle is the screen where the stimuli were presented during the test, side by side. The choice areas for the stimulus located on that side are represented in green and purple. (*b*) Representation of the choice criteria. Cumulative choice for one stimulus was scored every time the chick entered the choice area with two-thirds of its body (including the head).

The apparatus consisted of a triangular plexiplat arena (figure 2*a*), with white walls (height: 20 cm) and white floor. One side of the triangle (60 cm) was occupied by a computer screen where the stimuli were presented. At the beginning of the test, one chick was individually placed in the corner opposite the screen (starting corner) at a distance of 60 cm from the screen. The screen was divided into two halves by a white plexiplat wall (height: 20 cm; length: 15 cm, thickness: 5 mm). Stimuli (§3.3) appeared serially each in one half of the screen, where they stayed for three consecutive minutes. During this time, the chick was free to move within the arena and approach either stimulus. At the end of the 3 min, the subject was temporary removed from the arena, for few seconds and placed back in the starting corner; the stimuli swapped their position on the screen (the stimulus that had occupied the left part of the screen during the initial phase was then presented in the right part, and vice versa). After this positional change, stimuli were presented for three more minutes. The initial position of the stimuli was randomized between subjects. The whole-test session (6 min) was recorded with a videocamera placed above the arena and chick's approach behaviour was coded online minute by minute using the software Cyclic Timer and later scored offline by a naive, independent observer.

The apparatus was virtually divided into a no-choice area and two choice areas. The overall permanence inside each choice area was obtained by computing and adding the time spent in that area on each visit (from the moment the chick entered the area with its head and two-thirds of the body (figure 2*b*) and until it exit such area). See the electronic supplementary material, for a video extract of the free choice test. Latency to choice was measured as the time spent in the no-choice area before choosing a stimulus for the first time. The chick was then returned to the hatchery where it was kept in darkness until all the three individuals of the triplet completed the test session and were therefore ready to undergo the social rank evaluation.

### 3.6. Evaluation of social rank (pecking order)

The procedure was developed in our laboratory and previously employed to rank chicks [46]. Once tested individually, all three chicks of a same group were put together in a cage with white plexiplat walls (28 × 40 × 30 cm), provided with water ad libitum, and left free to move and interact for 15 min. The entire duration of this period was video recorded by a camera positioned above the apparatus. Pecking interactions among the three chicks were coded offline. The behavioural response consisted of the number of pecks received from, and directed towards, each other chick during the 15 min. The raw number of pecks emitted and received was recorded. Aggressive pecks, such as those directed towards the head or body of other chicks, were considered. The three chicks were then reared together in a cage with unlimited food and water in a room with controlled temperature (28–30°C) until day 5, when all chicks were donated to local farmers.

### 3.7. Alternative stimuli and further controls

If chicks exhibited no choice with the stimuli of the first study, we planned to use stimuli designed to stress more the interaction among the two agents. Specifically, the same agents (coloured squares) would move so as to make the aggressive interaction more salient: agent A would push agent B away, and agent B would not move to approach agent A after being hit. The two squares would be individually presented after the interaction, making sure that both are self-propelled and that their total amount of movement and time of appearance on the screen are balanced. Nonetheless, these stimuli cannot be as controlled as our first stimuli in terms of equivalence of movement of the two agents.

## 4. Results

### 4.1. Sample

Based on the sample used in previous studies with similar paradigms, [25,30,47], we decided for a total sample size of 200 chicks (100 per condition, among which 50 were males and 50 females). We performed an *a priori* analysis with the software G-Power, to assess the maximum power that could be reached with this sample size. We expected a small to medium effect size, and we performed the analysis considering a small effect size (*f*^2^ = 0.05; *α* = 0.05). Given these parameters, the power that could be reached with 200 individuals is 75%. Considering that animal studies reach a reduced power for both small and medium effects (respectively, around 13–16% and around 40–47%) [48], we were satisfied with these predictions.

Chicks not responding during the test phase were removed from the data analysis (total number of subjects tested *n* = 421; removed subjects *n* = 207, females: 36%); this included subjects which did not move from the starting corner of the apparatus for the entire duration of the test (*n* = 196) and chickens displaying high stress levels (the animal emitted sustained distress calls during the entire duration of the test; *n* = 11). No other exclusion criteria (i.e. sign of illness, movement difficulties, external events compromising the collection of data such as sudden noises or vibrations, drop of temperature during the experiment, failure of electricity, etc.) were met. Subjects removed were replaced, in order to reach the predicted sample size. Data collection ended when the predicted sample size was reached (*n* = 214; females: 47%).

### 4.2. Choice index

Choice index (CI) used in the analysis, calculated as:

$$\mathrm{CI}=\frac{{\mathrm{time}}_{B}}{\begin{array}{c} {\mathrm{time}}_{B}+{\mathrm{time}}_{A} \end{array}}.$$

A CI of 0 signifies choice of the aggressor only. A CI of 1 means choice of the victim only, and a value of 0.5 means equal amount of time spent next to both agents.

### 4.3. Coding validity

To assess the validity of the coding, data from around 30% of the subjects (both free choice task and pecking order test) were re-coded by a second coder, specifically trained and blind to the condition and the expected results. We performed a correlation analysis on the two codings, to assess inter-coder reliability, using Cohen's *K*. All indices showed a good reliability (free choice task, weighted-*k* = 0.98; pecks given, weighted-*k* = 0.80; pecks received, weighted-*k* = 0.82). The statistical analyses planned during stage 1 are reported in table 1.

**Table 1.** Hypotheses and planned statistical analyses, approved in stage 1 registered report.

question	hypothesis	sampling plan	analysis plan	interpretation given different outcomes
1. Do chicks attribute different roles to the perceived characteristics of the motion of artificial agents and show a preference for a specific role?	(H1) newborn chicks can discriminate roles from observed interactions, and (H1b) show a stronger preference for the approacher over the repulser agent in the experimental versus control condition	based on statistical considerations and previous literature, sample size is set to 200 individuals (100 per conditions—50 males and 50 females)	a logistic regression analysis will be performed to verify the effect of the manipulation, considering the CI as the dependent variable, and as factors, the condition (control versus experimental) (H1 and H1b)	1. A null coefficient for the condition would mean no difference in the CI between the control and the experimental condition (H1 and H1b not supported)
		we computed a power analysis using the software G Power	to verify H2, we considered the interaction between the agent's role and the chicks' sex (male versus female)	a positive coefficient (*p* < 0.05) represents a higher CI in the experimental compared to the control condition (H1 and H1b supported)
		we used a linear regression model, and considered two contributing factors (condition, sex*group interaction)	significance is conventionally set at *p* < 0.05	
		power was calculated and resulted to be around 70%, for an effect as small as *f*^2^ = 0.05 (*α* = 0.05)	a binomial test will be conducted in both conditions to assess whether the CI drops to chance level (= 0.5), and a *χ*^2^-test will be used to compare the frequency of choices in both conditions	a negative coefficient (*p* < .05) represents a lower CI in the experimental versus control condition (H1 supported, H1b not supported)
2. Is there a sex difference in chicks' behaviour and preference?	(H2) the sex of the chicks influences the preference for a specific agent (as measured by the CI)			2. A coefficient different than zero in the condition*sex interaction (*p* < 0.05) indicates an effect of the sex, according to the condition (H2 supported)

### 4.4. Hypotheses testing

The hypotheses were tested using a logistic distribution (generalized linear model), considering as the dependent variable the time spent by the subjects next to the target stimulus (the ‘approacher’, in the experimental condition, the second figure moving in the control condition), as measured by the CI (table 2). The model included, as predictors, the condition (control versus experimental) (H1 and H1b) and its interaction with the sex (condition*sex) (H2). We did not find a statistically significant effect of condition (*b* = 0.18, s.e. = 0.33, *p* = 0.58) nor a statistically significant interaction effect between condition and sex on the CI (*b* = −0.26, s.e. = .39, *p* = 0.51).

**Table 2.** CI values (mean and s.d.) for each condition and divided by sex.

	control		experimental	
	males	females	males	females
CI mean (s.d.)	0.57 (0.37)	0.44 (0.41)	0.56 (0.40)	0.49 (0.42)

### 4.5. Exploratory analyses

As planned in the registered report, we ran an exploratory analysis on pecking order and its potential influence on the chicks' preference. We used the raw number of pecks given and received by each subject, in order to differentiate between subjects having a very high-peck frequency and subjects having a very low-peck frequency. We tested a logistic mixed model, with a three way interaction between pecks’ frequency, their direction (received/given), and the condition (experimental/control) predicting the CI as the dependent variable; the model included the random effects of the subject and triplet. The interaction was not significant (*b* = 0.10, s.e. = 0.53, *p* = 0.85).

A second exploratory analysis on choice latency was tested, using as predictors the experimental condition and its interaction with pecking order (linear mixed model). In the model, we included the random effects of triplet and subject. No significant effect emerged (*b* = −20.88, s.e. = 21.28, *p* = 0.31).

### 4.6. Additional exploratory analyses

In addition to the planned analyses, we checked for the existence of an eventual side bias, which might have had an impact on the results, despite our efforts to avoid it through a thorough experimental design. First, we tested whether chicks generally preferred moving towards one or the other side of the apparatus. To do so, we calculated a side bias index (SB) using the following formula^1^:

$$\mathrm{SB}=\frac{L_{\mathrm{tot}}}{L_{\mathrm{tot}}+R_{\mathrm{tot}}},$$

where *L*_tot_ is the total amount of time spent next to the stimulus on the left side of the screen, and *R*_tot_ is the total amount of time spent next to the stimulus on the right side of the screen. A non-parametric test (Wilcoxon–Mann–Whitney) showed a general bias towards the right stimulus (*M* = 0.44, s.d. = 0.41, *V* = 9455, *p* < 0.05), not depending on the condition (*M*_exp_ = 0.42, *M*_cont_ = 0.45, *W* = 5913, *p* = 0.58). Then, we checked for an effect of the stimulus position on the choice in the two conditions. We performed a logistic regression, considering the CI as the dependent variable, and both the condition (experimental versus control) and its interaction with the position of the target stimulus as the predictors. As for the previous models, the simple effect of the condition was not significant (*b* = 0.36, s.e. = 0.21, *p* = 0.24). Interestingly, the interaction between the position of the target and the condition was significant (*b* = −0.67, s.e. = 0.31, *p* < 0.05). To further explore this effect, we used a non-parametric test (Wilcoxon–Mann–Whitney) to compare CI in each condition with chance. Specifically, in the control condition the CI did not significantly differ from chance (corresponding to a CI = 0.50) whether the target (agent B) was on the left (*M* = 0.48, s.d. = 0.47, *V* = 1904, *p* = 0.66) or on the right side of the screen (*M* = 0.55, s.d. = 0.83, *V* = 2043, *p* = 0.30). In the experimental condition, the CI did not significantly differ from chance when the target was on the left side of the screen (*M* = 0.46, s.d. = 0.47, *V* = 1533, *p* = 0.40), but was significantly higher than chance when the target appeared on the right side of the screen (*M* = 0.62, s.d. = 0.45, *V* = 2478, *p* < 0.05).

## 5. General discussion

The present study aimed at exploring the early emergence of the ability to discriminate the social role of conspecifics by observing their interactive behaviour, and prior to social experience, in the domestic chicken. The research further considered the sex of individuals as a potential factor impacting such an ability. Neither males nor females showed a preference for one over the other agents in the experimental nor in the control condition. Moreover, pecking order did not affect chicks' preference for one or the other agent. Additionally, an exploratory analysis did not reveal an effect of the condition, nor of pecking order, on latency to choice. Thus, the data are not in line with our hypotheses and we cannot confirm the existence of an innate component of the ability to discriminate third parties’ social role from observed interactions in the newborn domestic chicks. Nevertheless, neither could we rule out the possibility that chicks could discriminate between the two agents, despite not showing a clear preference for one. In fact, it would be theoretically possible that, while being able to attribute different roles to the two agents, chicks did not have a preference for either role, thus, choosing randomly between them. With the present data, though, it is hard to disentangle the two possibilities. Thus, our results could be either driven by the absence of a strong preference for two equally desirable, yet different, roles, or by a lack of understanding of the behavioural difference between the two agents. As argued more in depth later in this section, the stimuli could have been too complex to be easily discriminated, or been strongly constrained by some other factors (see the next paragraph on the lateralization of cognitive abilities). Alternatively, the discrimination of two agents based on their behavioural interactions might be an ability that develops later in life, thanks to the direct experience of social interaction with conspecifics. We further discuss this option in the final paragraphs of the manuscript.

In addition to the main hypotheses testing, we ran an exploratory analysis on side bias, which showed a significant interaction between the condition (*experimental* versus *control*) and the side (*left* versus *right*) in which the target agent (the ‘approacher’) appeared, revealing an interesting behavioural pattern: a significant preference for agent B (the ‘approacher’ agent) emerged in the experimental condition, when the agent was presented on the right side of the apparatus. This suggests that the discrimination abilities and the display of a preference for one or the other agent could be already present in the newborn chick, but they might be too feeble to show a clear effect and might be strongly constrained by the brain lateralization of cognitive and social skills typical of the species. Specifically, in the experimental condition the CI was significantly above chance when the target agent (agent B, the ‘approacher’) appeared on the right side of the screen, while it dropped to chance when it appeared on the left. The choice for agent B, though, was at the level of chance in the control condition, both when it appeared on the right and on the left side of the screen. In other words, presenting agent B on the right side of the screen increased the preference for that agent, but this occurred selectively for the experimental condition. These findings suggest that lateralization might play a role in discrimination and/or preference for one agent over the other. In fact, lateralization of cognition is widely spread in avians, including the domestic chicken [49–51]. Several studies illustrate the differential role of the two hemispheres in regulating and controlling different cognitive abilities in chickens: for instance, the ability to recall a stimulus from its position is mainly controlled by the right hemisphere [50], while recalling it from its object-specific cues is mainly a left hemisphere task [50–52]. Additionally, the left hemisphere controls the initial memory encoding [52]. Relevant to the present work, social cognition is also strongly lateralized in the chicken brain: specifically, the right hemisphere (left visual hemifield) is responsible for the discrimination of novelty detection and of familiar versus unfamiliar individuals, while the left hemisphere (right visual hemifield) is responsible for the differentiation of familiar objects (thus, potentially, familiar social agents) by specific object-related cues [52–55]. Although we are not aware of any published study investigating the ability to discriminate between two or more familiar individual in chickens, it is plausible that this skill is performed by the left hemisphere, based on the literature cited above [52–55]. Additionally, Andrew *et al*. [56] reported that the left hemisphere (right visual hemifield) is responsible for inhibiting the behavioural response to an alternative stimulus. In the light of this, we can cautiously interpret our results as follows: when agent B appears in the right half of the screen, it is processed by the chicks' right eye (thus, its left hemisphere). The left hemisphere both processes object-specific characteristics of familiar individuals (in this case, agent A and B), and has an enhanced capacity of inhibiting the response to the alternative stimulus (agent A, presented on the left side of the screen). Thus, it might be easier to recognize, and choose, agent B when it appears in the right spatial (and visual) hemifield, compared to when it appears in the left spatial (and visual) hemifield. Indeed, when agent B was on the right side, and in the experimental condition only, chicks chose it significantly more than chance, mirroring children [32–35,40] and adult chickens [43] and in line with our first hypothesis. On the contrary, when the agent was on the left side, performance dropped to chance. Choice was at the chance level in the control condition (no difference in agents’ roles), both when agent B appeared on the right or on the left side of the screen, suggesting that in this condition the two agents had equal valence for the subjects. Importantly, we based this hypothesis on the fact that chicks were mainly pointing their beaks straight forward, towards the computer screen, during the choice; thus, they would look at the right stimulus with the right eye, and at the left stimulus with the left eye. However, as this was not the main goal of the study, we did not formally encode the eye used to observe the stimuli. We think that a more formal analysis will be needed to confirm our hypothesis. Even so, these findings open the path to new interesting questions on the development of social abilities and their biological constraints. Future research should deepen these issues. For instance, the use of eye-occlusion techniques would allow us to check the actual impact of the eye of use in stimuli discrimination and preference. In such a case, it would be important to consider eye-occlusion both during the familiarization and the free choice phases, so to disentangle whether the lateralization impacts the encoding (familiarization phase) or the retrieval (free choice test) of the information, or both.

Some limitations of the research are worth commenting on. First, while several studies demonstrated that newborn chickens do attribute agency to self-propelled geometrical objects [30], the use of geometrical animated agents might hinder chicks' ability to understand the different roles of the agents appearing in the video sequences we used. In fact, to better control for the stimuli's characteristics, the social agents we used were coloured squares, and moved rigidly, as a whole. A more naturalistic expression of aggressive behaviour in chickens would imply relative motion of part of the body, as in pecking directed at a conspecific. Natural movements are therefore visually very different from the interaction depicted in our stimuli (where agent A moved towards and hit agent B). Thus, it could be that newborn chicks struggled to interpret the agents' movement. This could also help explain the high number of chicks that did not choose between the two stimuli and had to be excluded from the analysis. It is not unusual that a high number of chicks fail to respond to the tests (see for instance: [57]); in particular, the high complexity of the stimuli used in the present set-up might have significantly contributed to a high rate of subjects not choosing either of the stimuli. The use of a more naturalistic representation, for instance a recording of a real interaction between two newborn chicks, could be a useful tool to check for this possibility and increase the number of chicks engaging in the task. Nonetheless, it would also pose additional issues: the recording of a real interaction cannot be controlled for other confounding factors, such as the total amount of movement of the two agents, or the potential effect of their specific physical features in shaping the preference for one or the other. Additionally, while it is known that newly hatched chicks can easily discriminate between a familiar and an unfamiliar conspecific [58,59], the literature still lacks studies demonstrating that they are able to discriminate between two familiar conspecifics, without physically interacting with them, while they can easily discriminate familiar agents by their colour [30]. Thus, we believe that future studies should carefully consider both options, acknowledging the strengths and limitations of each. On another note, following the suggestion of one reviewer, we checked *a* *posteriori* the luminance of our stimuli (between 45 and 47.5 cd × m^2^), as the flicker fusion frequency (i.e. the frequency at which a flickering image is no longer perceived as such, but is seen as continuous instead) might depend on it (see [60], table 1 and figure 2). According to Lisney and colleagues [60], stimuli of around the same luminance as ours would need a 65–70 fps, which is just above the refresh rate we had (60 fps). On the other hand, Rubene *et al*. [61] found a lower flicker fusion frequency for even higher luminance levels (around 55 fps per 120 cd × m^2^). Thus, it is not clear whether our 60 fps videos (cf. [25,30,47]) would pose a problem to the perception of a continuous stimulus. However, in their research on newborn chicks’ preference for self-propelled movement, Mascalzoni *et al*. [30] found a very clear effect in the experimental versus the control condition, which might bring good evidence that a refresh rate of 60 fps does not severely affect the correct perception of motion. Nonetheless, future work shall consider and explore this factor more carefully.

Another critical point is represented by the timing of the pecking order test. Dominance was tested to check whether an innate predisposition to a certain social rank could be linked to chicks' discrimination abilities or preferences. Being constrained by the necessity of testing socially naive chicks, the dominance had to be measured after the free choice test. Dominance testing, though, only allowed us to measure social rank as determined by chicks’ aggressive interactions during a 15 min timeslot. During video coding, we noticed that the aggressiveness of some individuals changed throughout the test, with some of them starting as more aggressive, and becoming less aggressive as the other chicks started to respond to their pecks, and *vice versa*, initially submissive (i.e. receiving many pecks and not reacting) individuals could become more aggressive and dominant by the end of the observation. As a consequence, this measure might be more appropriate to understand how the hierarchical structure is dynamically built, than to measure the *a priori* predisposition to a social role (which can either be expressed or suppressed once social interactions start).

Another interesting observation arises from the work by Rogers & Workman [62] who suggested that transitive inference, while still present in dark-hatched chicks, would be enhanced in light-hatched chicks. Our chicks hatched in the dark to limit their social (and visual) experience of conspecifics prior to the experiment; this could have caused a lower ability to perform logical reasoning, thus affecting our results. A strategy to avoid this issue could be to design and use a hatching machine with individual compartments for eggs, thus preventing the newborn individuals from experiencing sociality before the experiment, while at the same time allowing a normal development of their lateralized cognitive abilities (for studies using individualized hatching compartments, see [26,63]). In addition, the present findings suggest that the ability to discriminate social roles from observed interaction, and/or the preference to approach an individual with a specific social role, which is present in the adult chicken [43], might emerge later in life, or might be facilitated by the first social experiences, as found in human infants between four and eight months of age (cf. [32]). Thus, future studies should also explore the role of social experience in the development of social preference.

## 6. Changes to stage 1 registered report

In this section are briefly described the differences with the stage 1 report, and the reasons behind them. Firstly, we decided to cite a recent paper [32] using the same paradigm and stimuli in the introduction, as it fits and strengthens the theoretical background of the study. Importantly, following the terminology used by Geraci *et al*. [32], we have decided to update the terminology used in this manuscript, to facilitate comparisons. Specifically, relative to agent B we substituted ‘chased’ with ‘approacher’, and relative to agent A we substituted ‘chasing’ with ‘repulser’. Despite this change, the main concepts, as well as the hypotheses and the theoretical background, are not altered. Secondly, we ran an additional analysis, reported above in §4, under the section *Additional exploratory analysis,* §4.6. In consideration of the existing literature on side bias and lateralization in the domestic chicken, a formal analysis exploring this possibility seemed suitable. Indeed, it revealed interesting results, opening the path to future research. Another important note concerns the alternative control study we proposed in the stage 1 report, described at the end of the *Material and methods* section. We initially proposed the use of different videos depicting the interaction between the two agents in a more extreme way, in the case our subjects would not have shown a clear preference for one of them in the experimental condition. Whereas the main hypothesis was not confirmed, the results emerging from the side bias analysis do show the existence of the ability of newborn chicks to discriminate the two objects from their interactions, although constrained by the spatial disposition of the stimuli. We acknowledge that this only partially supports the decision not to run the additional experiment. Unfortunately, a series of events hindered the possibility to perform it; the COVID-19 pandemic and multiple aviary flu epidemics, in addition to ministerial restrictions to animal testing, consistently slowed down data collection. Thus, after thorough considerations, and in light of the additional results reported in the manuscript, we decided to avoid the risk of not being able to finish the additional data collection.

## Data Availability

Following in-principle acceptance, the approved stage 1 version of this manuscript was pre-registered on the Open Science Framework at: https://doi.org/10.17605/OSF.IO/WSHU8 [64]. This pre-registration was performed prior to data analysis. All data and the R script used for the formal analysis, together with a video sample of the Free Choice Task, are uploaded in the same repository. The data are provided in the electronic supplementary material [65].

## Footnotes

1 Following the formula, a right-side bias is indicated by an index SB < 0.5, a left-side bias by and index SB > 0.5, while SB values around 0.5 indicate no side bias.
